# Building peer information exchange networks to improve child and adolescent TB care in Sub-Saharan Africa

**DOI:** 10.5588/pha.25.0036

**Published:** 2025-12-03

**Authors:** R. Mande, S.D. Berger, B. Moore, P. Thekkur, J.P. Dongo, J. Doyle, J. Harris, S.M. Graham, R.A. Dlodlo

**Affiliations:** 1 International Union Against Tuberculosis and Lung Disease, Kampala, Uganda;; 2 International Union Against Tuberculosis and Lung Disease, Paris, France;; 3 U.S. Centers for Disease Control and Prevention, Division of Global HIV and TB, Atlanta, GA, USA;; 4 Melbourne Children’s Global Health, University of Melbourne Department of Paediatrics and Murdoch Children Research Institute, Royal Children’s Hospital, Melbourne, VIC, Australia.

**Keywords:** child and adolescent TB, peer learning networks, capacity building

## Abstract

The Sub-Saharan Africa Regional Child and Adolescent TB Centre of Excellence (COE) was established in 2019 to address gaps in child and adolescent TB care in the region. The COE promotes the south-to-south exchange of best practices and innovation through virtual and in-person engagements, including webinars, workshops, and annual meetings. The COE’s training efforts, including an interactive curriculum, have strengthened capacity that has increased TB detection and enhanced knowledge among health professionals.

TB remains the world’s leading infectious cause of death even though it is preventable and curable; it is common in children and young adolescents (<15 years).^1^ Almost all TB-related deaths in children and adolescents occur in those who do not receive adequate TB care because they are not detected.^2^ Furthermore, many children with TB represent missed opportunities for prevention as global implementation of contact investigation and TB preventive treatment remains low. Overcoming these challenges in child and adolescent TB requires targeted interventions that can continuously and flexibly evolve alongside the needs of the population. One such effort is the Sub-Saharan Africa Regional Child and Adolescent TB Centre of Excellence (COE). The COE was established by the International Union Against Tuberculosis and Lung Disease (The Union) in collaboration with the U.S. Centers for Disease Control and Prevention (CDC) in response to a regional meeting with Ministries of Health (MOH) in Kampala, Uganda, in 2019 focused on ways to improve the management of child and adolescent TB. The goal of the COE is to advance the Childhood TB Roadmap^3^ by capacitating national leadership, bridging the policy–practice gap, and fostering partnerships to improve and expand interventions to end TB in children and adolescents. The COE was envisioned primarily as a virtual platform promoting the south-to-south exchange of best practices, collective problem-solving, and discussion of relevant research findings. Nine countries (Eswatini, Ethiopia, Kenya, Malawi, Mozambique, Tanzania, Uganda, Zambia, and Zimbabwe) became founding members of the COE and were joined in 2022 by Cameroon, Lesotho, and Rwanda (Figure 1). Each country identified two representatives to serve as members of the COE, with most members coming from the National TB Program (often the Child TB Focal Point), MOH, or another relevant department. An Advisory Committee of representatives from leading multilateral, academic, and non-governmental organisations was established to guide the COE and identify opportunities for collaboration.

**FIGURE 1.**
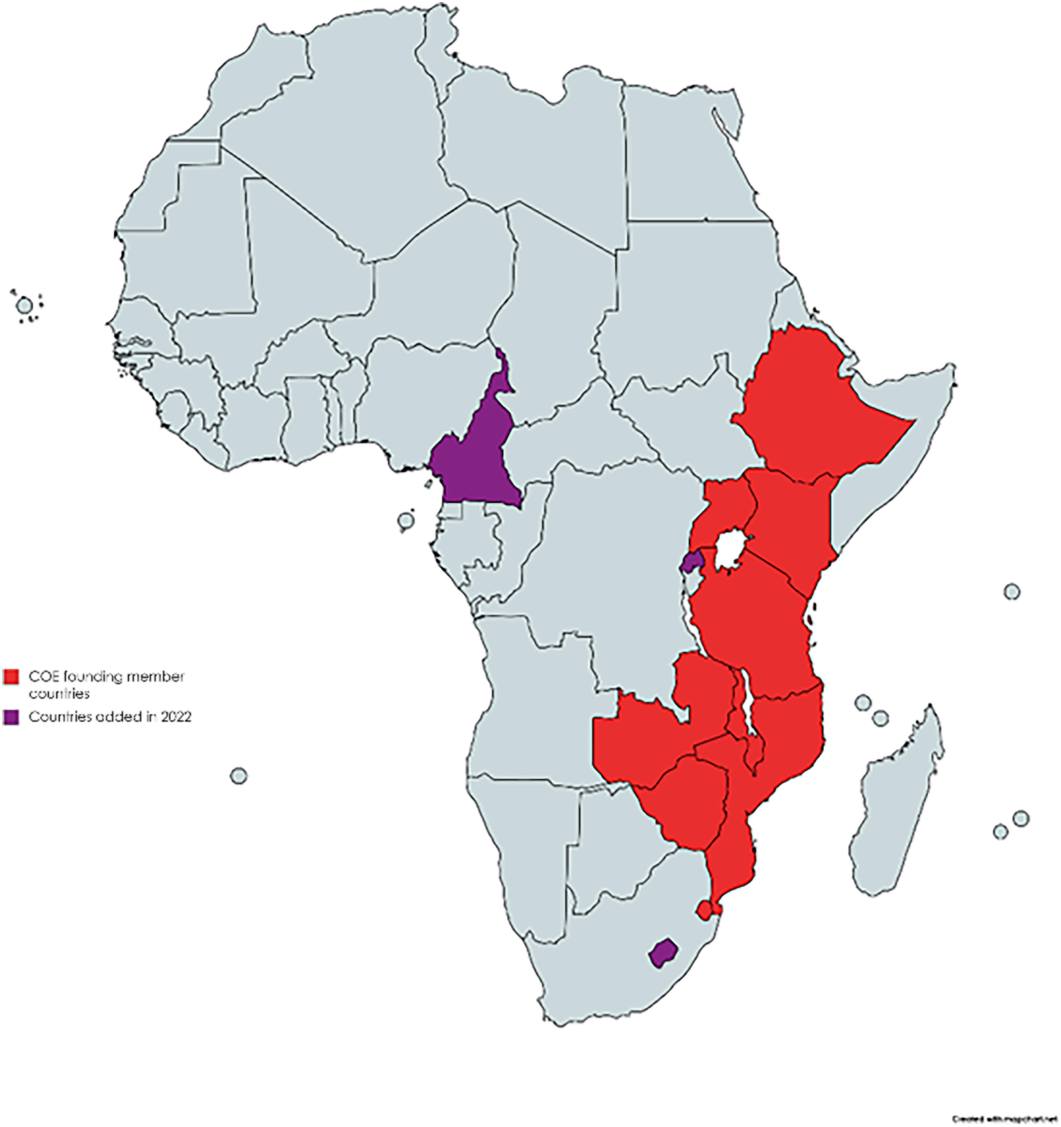
Member countries of the Centre of Excellence.

The virtual nature of the COE enabled continuity of activities amidst travel restrictions during the COVID-19 pandemic. The findings of a landscape assessment undertaken in 2021^4^ and frequent engagement with members through regular meetings and interactive polls helped to customise and adapt COE offerings in line with the needs and preferences of the members. The COE has offered a range of activities and services to member countries since its inception, including in-person and virtual meetings, technical webinars, tools and training materials, in-depth workshops, and a comprehensive training curriculum for management of child and adolescent TB (Figure 2).

**FIGURE 2.**
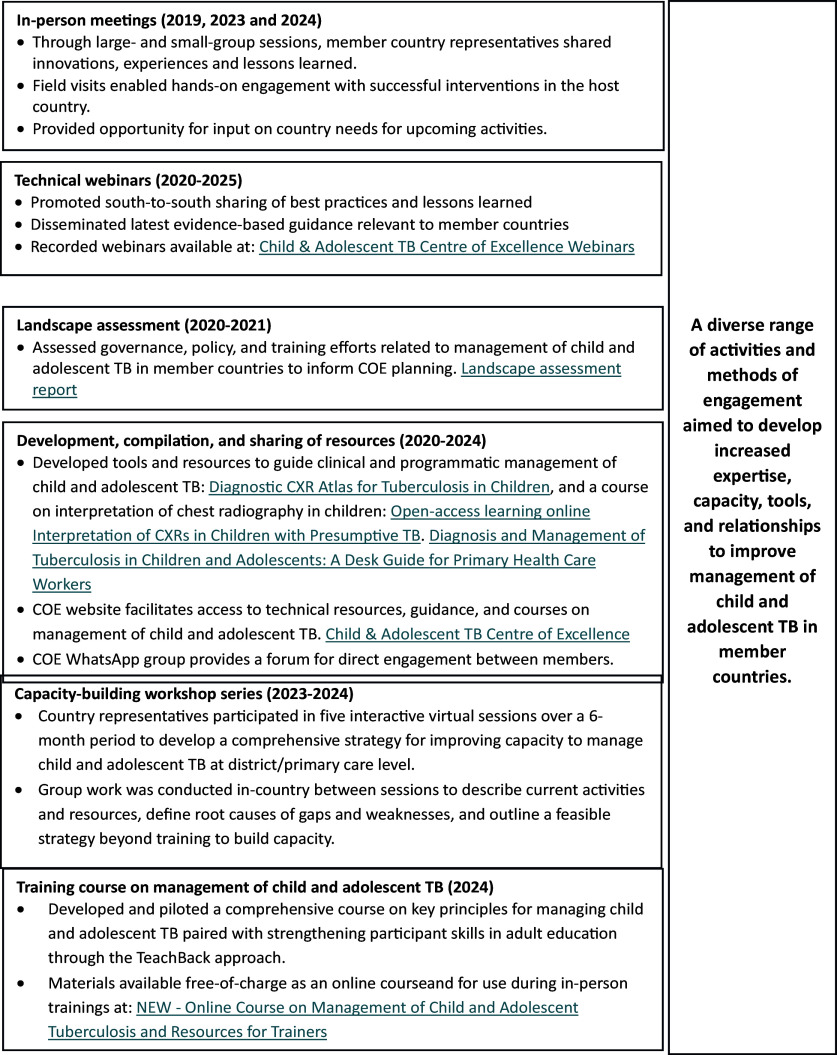
Child and Adolescent TB Centre of Excellence activities and resources.

## ANNUAL MEETINGS

The in-person annual meetings were hosted by a COE member country and attended by COE members, Advisory Committee members, and staff from the host MOH. These meetings provided opportunities for networking and sharing ideas paired with structured field engagements, promoted hands-on learning, and facilitated small group discussions about specific technical challenges that were difficult to achieve in virtual engagements alone. COE members shared experiences and engaged in group work and breakout sessions focused on common challenges, allowing members to share strategies and brainstorm solutions. Field visits provided opportunities to observe successful interventions in the host country that could be adapted to their respective settings. Such hands-on experiential learning and collaborative problem-solving during these visits further enriched the experience of in-person meetings. One of the COE representatives who attended the in-person meeting in 2023, which included field review of child-friendly TB diagnosis, said,

These meetings enabled us to interact with other stakeholders, learn from other countries’ experiences and broaden our views on TB diagnosis using Xpert on stool specimens. With this, we were able to develop standard operating procedures to implement TB diagnosis using stool among children in our country.

Technical Webinars and Clinical Case Discussions

In addition to discussions at the annual meeting, routine technical webinars showcased the latest research, local innovations, and practical approaches to implementation with facilitated discussions involving a broad audience of participants on the topics selected by the members. The webinars have received positive feedback, as one paediatrician mentioned,

The programmatic experience shared during the webinar on ‘Nutrition and tuberculosis: considerations for national programs and child and adolescent tuberculosis’ triggered multiple ideas and thoughts to incorporate TB screening at the various entry points where children with malnutrition seek care.

Capacity Building and Training Curricula

One critical gap highlighted by members through discussions and polls and at annual meetings was the absence of updated, relevant, and interactive training curricula on child and adolescent TB for health care workers. The COE addressed this gap by engaging experts in paediatric TB and adult pedagogy to develop a comprehensive in-person course on child and adolescent TB that was piloted in Lesotho in 2024. The course reviews principles in paediatric TB through an interactive approach while strengthening participants’ skills in adult education by introducing the CDC TeachBack approach and reinforcing facilitation skills of health care workers.

Following the pilot, The Union and CDC revised the training materials to incorporate feedback. The revised training package, based on insights from the pilot, also served as the foundation for developing an online course on child and adolescent TB. Both the online course and a resource package for in-person training is available free of charge on The Union website. Lesotho rolled out the in-person curriculum at sub-national trainings across half the country in 2024, ultimately training 240 district-level health professionals. The trainings were appreciated by trainees, as a participating doctor commented,

The training has massively strengthened my understanding and ability to diagnose and manage TB in children and adolescents. It has also enhanced my confidence in educating other health care workers about TB care and prevention.

An increase in the proportion of children among new TB diagnoses was observed following these trainings in Lesotho. For example, comparing data from March and April 2024 to March and April 2025, the Lesotho Ministry of Health reported that notifications among children as a proportion of overall notifications rose from 3% and 4% in 2024 to 7% and 11% in 2025, respectively.^5^

While many elements of the COE have been successful, we did face some challenges. In-person meetings could not be held from 2020 to 2022 due to the COVID-19 pandemic, which limited interactive, hands-on engagement. We also learned that multi-country, multi-session, facilitated virtual workshops were demanding to execute, given competing priorities, schedules, and time zones among both members and resource persons. Careful consideration for what must be directly facilitated (as opposed to completed independently) and what amount of additional work is required of participants is critical to ensuring engagement and completion of deliverables.

In conclusion, south-to-south learning platforms facilitate networking among peers across countries and enable exchange of evidence and support collective problem-solving of common challenges. While virtual platforms are beneficial, in-person engagement remains crucial to take on larger or more complex topics and to build and reinforce relationships. Identifying opportunities for informal, direct engagement in the virtual space is important to maintain connection and ensure the network is accessible and can be used in real-time.
